# "The Hospital" Nursing Mirror

**Published:** 1897-09-25

**Authors:** 


### "The Hospital" Nursing Mirror.

Being the Nursing Section of "The Hospital."

[Contributions for this Section of "The Hospital" should be addressed to the Editor, The Hospital, 28 & 29, Southampton Street, Strand, London, W.C., and should have the word "Nursing" plainly written in left-hand top corner of the envelope.]

### News from the Nursing World.

### TYPHOID AT THE ROYAL FREE HOSPITAL.

An inaccurate and misleading statement has been printed and circulated respecting an outbreak of typhoid fever amongst the nurses at the Royal Free Hospital. We have made inquiries of the authorities, and are glad to be able thus to reassure those whom the alarming rumour may have reached. Cases of typhoid fever have occurred in the hospital from some cause not yet ascertained, but only one nurse has been the sufferer, and she is now nearly convalescent. The other members of the hospital staff who have suffered have been three house-surgeons and the housekeeper, and all of these are convalescent. The report as to a fatal case amongst the nurses is entirely without foundation. The hospital authorities have been most thorough and zealous in their search for a cause for the outbreak, and so far nothing has been found within the hospital to account for it. From this, and also from the fact that no fresh case has occurred since August 12th, we hope that the infection was introduced from without, and will not be again introduced.

### AN AMATEUR NURSE.

The little children of Major Des Vœux, who were in Fort Gulistan during the recent attack, have become quite celebrated by their original description of the proceedings; and their nurse, Miss Teresa M'Grath, earned the admiration of all by the courageous and devoted manner in which she ministered to Lieutenant Blair when he was wounded. She attended on him quite regardless of the bullets flying around her.

### "AN INDIGNANT NURSING STAFF."

We have received a letter signed as above from the nursing staff of an infirmary, in which the complaint is made that a new superintendent of nurses directed one of the staff who had nearly completed her training to assist in a ward which was in charge of a junior nurse. The expostulation of the nurse, and a request to be moved elsewhere was disregarded, according to the account of our correspondent. We are asked for our opinion and advice in the matter. We much regret that friction should have occurred between the superintendent and her staff, possibly the result of misunderstanding, which, if the superintendent is wise, she will take steps to remove. At the same time, we cannot recommend revolt on the part of the nurses because they do not like the orders which they receive from their superior officer. According to facts, the nurse in question will soon have completed her training, and she is not bound to remain where circumstances are not congenial; but until she is free to leave the institution we cannot uphold her nor any other nurse in a revolt against their superintendent. If the superintendent acts in such a manner as to impede a nurse's progress in her profession she can always resign, and state her reason to the board of management for doing so. Most superintendents are the nurses' best friends, and something must be very definitely wrong if nurses feel compelled to combine against their superior officer. Even in extreme cases a courteous protest will remove reasonable objections, and, as a last resource, there is always the board of management to appeal to.

### COMMON SENSE.

The cudgels have been taken up for nursing as a profession in a very sensible manner by a writer in the *Spectator* for September 11th, one who describes herself as one who never had the slightest desire to take nursing up "as a calling" or otherwise, and who signs herself "An Old Woman" who is proud to count several professional nurses among her friends. She asks the pertinent question, "Why is it a 'danger' that 'a woman may take to nursing as she will open a shop or do typewriting?' If," she points out, "a woman has not the necessary faculty she will fail at either career; but supposing she has not a sufficient income, why should she not attempt a profession which, besides affording her bread and possibly providing for her old age, enables her to serve her country by saving life sometimes and curing pain often? You recognise that 'sentimentalism' is not what is wanted in a nurse; is it not equally true that what *is* wanted is honest devotion to duty, and may not 'duty' be fairly said to include keeping herself off the rates, whether of the parish or of her acquaintance? Is the ideal of a nurse to be a 'ministering angel' bent on self-sacrifice here and reward hereafter? or is it to be a thoroughly-trained member of a profession as necessary and as well worthy of payment as army, law, or medicine?"

### OBEDIENCE WITH ECONONY.

That the new regulations of the Local Government Board in respect to nursing would admit of a certain elasticity of interpretation was only to be expected, and also that there would be plenty of Guardians ready to ring all the variations possible was equally obvious. The Cardigan Board of Guardians have started the subject with a most frank declaration that they will take advantage of what elasticity is at their command. The chairman at a recent meeting, in explaining the duty before them of securing a nurse for the workhouse, commenced by saying they were not bound to get a nurse trained in a hospital, but it was necessary that she should be practically experienced in nursing. One of the Guardians inquired what was meant by practically experienced. The answer was that he must define that, as it was in the Order. Another Guardian explained that they would have to satisfy the Local Government Board. Yet another Guardian was cheered by the inspiration that if they had to appoint a nurse they might dispense with one or more of the other persons employed in doing the work of the workhouse. This question being entered into, the staff of four persons already employed was put in review, and it was proposed that the services of an antiquated and feeble person, who assisted in the house at a wage of one shilling per week, might be dispensed with; but this was objected to as improvident by some one else, as it was pointed out that if they took her into the workhouse, of which she was admittedly a proper inmate, they would have to pay her three shillings a week. After some plain speaking on the part of some as to the general conduct of those in authority in respect to the employment of this decrepit old lady, it was resolved to advertise for a nurse, not necessarily trained, but able to do all duties when required, at a salary of £16.

### NURSING IN SUFFOLK.

Great encouragement is being offered to district nursing in Suffolk. The Technical Instruction Committees of the East and West Suffolk County Councils offer seven scholarships annually to candidates recommended by the Suffolk Nursing Association, to enable Suffolk women to train as nurses. The Nursing Association provides the outfit, and defrays all other expenses incidental to the period of training, and guarantees the nurse when trained three years' employment at a salary of not less than 12s. a week. Seven candidates were trained last year, and are now employed or about to be employed, by the Association. By this, of course, it will be seen that the training required is of a minimum description. The candidates must be between 25 and 40 years of age.

### THE MEDICO-PSYCHOLOGICAL ASSOCIATION.

The next examination of the Medico-Psychological Association for their certificate in nursing and attending on the insane will be held on November 1st. October 4th is the last day upon which names of candidates can be received by Dr. Spence, Burntwood Asylum, Lichfield.

### THE ROYAL NATIONAL PENSION FUND FOR NURSES.

We are asked to announce that the Pension Fund has now invested funds to the extent of £350,000, a grand testimony of self-denial and providence on the part of nurses. At the last annual meeting the management were proud to report a sum which exceeded all expectations, amounting to rather over £311,000. To-day, only six months later, these invested funds stand at £350,000—an effective reply to those who when the fund was initiated denied women's power to combine, and keep to a provident scheme on a business basis.

### THE PROGRESS OF DISTRICT NURSING.

Progress is often stayed by a want of the true understanding of things. Had not Miss Walton Evans been present at the last meeting of the St. Asaph Board of Guardians, that body would probably have declined to lend their support to a local scheme of district nursing. It was urged that the farmers had to contribute largely to the cost of keeping the towns going, and it was not right that the rural ratepayers' money should be spent in that direction. Miss Evans pointed out that in the district where no nurse was employed that the Guardians had had to pay £20 in one year for nursing, and that not only would the Guardians save money by the establishment of a nurse, but that a generally healthier race might be the result through the education in hygiene the nurse could infuse amongst her patients. She pointed out that the infant mortality in the district she alluded to was very large, much larger than at Denbigh, which possessed a nurse. The result of her common-sense remarks was that the Guardians consented to contribute £2 2s. to the Nursing Association—not a very large sum, but still a substantial move in the right direction.

### THE NATIONAL SOCIETY FOR THE PREVENTION OF CRUELTY TO CHILDREN.

Many whose attention has been engaged by the affairs of this society will be interested to learn, according to a letter to the Press from the director of the society, the Rev. B. Waugh, that the executive committee will consider at its meeting, on October 11th, Lord Herschell's recommendations, and will report upon them to the council of the society at its meeting on November 9th, after which the decision of the council will be made public.

### NURSING THE TROOPS IN INDIA.

Hospital arrangements are being vigorously or ganised for the Indian frontier. There are 66 field hospitals available for use. At present there are four in the Malakand district, two at Tochi, three at Peshawur, and one at Kohat, all British hospitals. There are also native hospitals besides, and native hospitals and depôts elsewhere. A good many sisters have gone to the front already, and more doubtless will soon be on their way. Twenty-three hospitals have been mobilised in all, and two British and two native hospitals have been established with field medical stores. Thirty-one of the Army Medical Staff have been employed, and 65 of the Indian Medical Service are to be in readiness also.

### A HOLIDAY FOR NURSES.

It is the pleasant custom of the board of management of the Bradford Infirmary to organise an annual excursion for the nurses. This year the party wended its way to the Ilkley and Bolton Woods. The nurses went by train to Ilkley and had an excellent lunch there. They then drove in carriages to the woods, and spent the afternoon pleasantly in rambling in the delightful surroundings. After tea at Ilkley they returned to the infirmary. The matron and some of the medical staff accompanied the nurses.

### SHORT ITEMS.

The committee of the Truro Nursing Association have engaged Miss Yate as district nurse. She comes with excellent references, and has had ten years' experience in work amongst the poor. She was trained at the Westminster Hospital, and acted as superintendent to the Cardiff District Nursing Association previously.—The tender of a local contractor of £2,396 has been accepted for the erection of the proposed new nurses' home at Swansea in commemoration of the long reign.—We admire the determination which has been arrived at by the miners of Newbury Bridge in Wales to establish a district nurse in their midst, but we think it is a pity that their advisers should mislead them by such a statement as was made at a recent meeting, that two trained nurses could be employed at an expenditure of £40 a year.—The Local Government Board have concerned themselves with the nursing at the Westport Workhouse in Ireland, and desire the guardians to take immediate steps to appoint assistant nurses "of moral character and of sound mind" for the infirmary. The Westport paupers have been nursed by two untrained nurses, assisted by paupers, several of whom are of weak intellect.

### Practical Aspects of a Nurse's Life.

By Sister Grace.

### VI.—PROBATIONERS IN SURGICAL WARDS.

### Accidents and Emergencies.

You will now have acquired a fair knowledge of the routine of ward work, and be able to absorb more easily the knowledge presented to you. Surgical wards are more busy than medical, not that the work is always heavier, but it involves more movement; the amount of clearing away and continual tidying and cleaning up after dressings, constitutes a large item of the nurse's work, and of this she must not weary, for it is one which will endure till her last day in hospital. The changing of splints, the perpetual dressings and fomentations all make litter, which must be promptly and quietly removed. When a probationer first arrives in a surgical ward she is apt to feel despairing at the constant clearing away that is required, and the difficulty of keeping the ward in perfect order. Be careful to cultivate quietness and calmness of deportment, never allow yourself to be flurried; nothing inspires so much confidence among the patients as an unexcited demeanour. You will probably observe that the sister is calm and quiet, and you will learn to put trust in that quiet deportment, and endeavour to imitate it. Avoid all temptation to slovenliness; it is a snare to the new probationer to neglect small duties, because she has a little more to do than is easy to accomplish in the time at her disposal, her excuse being "I really had not time," or, "When that fracture came in I was so busy that I quite forgot." It is the sign of a good nurse never to forget small duties in the absorbing interest centring round more striking ones. Many nurses, otherwise reliable, are of little use in accident and operation wards from this tendency to be flurried in emergencies, and from their inability to put on sufficient steam to do double the usual amount of work in a given time.

The daily routine must go on, and yet time be found to receive accidents, wash patients brought in as emergencies, accompany the sister, perhaps, to the operating theatre, &c. If you can learn to do all this quickly, quietly, and cheerfully, you are in a fair way to make a good nurse. All this takes time to learn, but don't be content till you realise the ideal depicted by a house surgeon of the accident sister of his hospital; "I believe, Sister, that if six accidents arrived and the ward was on fire simultaneously, you would not be disturbed out of your usual calm." Emergencies, not in themselves perhaps very serious, have a great tendency to arrive at inconvenient moments, when dinner is being served or you are cutting bread and butter for tea, or are doing some important dressing. The first thing to do is generally feet washing, if nothing further, and all appliances must be put ready for the surgeon. You will be taught by the sister all that is required for different accidents, and see that you restore all articles after use to their proper places; forgetfulness in this may cost a life.

### Fractures.

Besides supplying all splints, &c., which you know to be required, never omit to have the dressing basket at hand, for you cannot be sure what alteration the surgeon may need to make, and nothing is more annoying than to have a nurse continually flying off to find things that have been forgotten. Have screens ready to be drawn round the bed, and when the surgeon comes remove all bedclothes but one blanket; have a towel ready for the sister's use in case the doctor wishes all coverings removed. The splints for compound fractures are covered with some mackintosh material. It is well to accustom yourself to the names of all the splints in general use. In cases of compound fracture dressings and antiseptic lotions will be required, and never forget to have hot bottles in readiness, however slight the accident may be.

### Hæmorrhage.

Always have mackintoshes at hand; it is wasteful and uncleanly to allow a mattress to be spoilt for want of a little forethought. It will be some time before you are called upon to receive and give first aid to serious accidents, such aid being rendered by the sister or staff nurse, but unforeseen circumstances may make it necessary for you to come to the front. If hæmorrhage occurs from a compound fracture, or, indeed, from any other cause, watch it carefully, and if it increases or continues to a serious degree send at once for the doctor, though he has probably seen it before; something has so altered the condition of the vessels as to cause the bleeding to increase seriously. Do not hurry away bloodstained cloths, put them out of sight, but where the doctor can readily see them, so as to judge of the amount of blood lost. Keep cloths soaked in carbolic over the injury until you have definite orders what further to do. Never wash patients whose condition is serious without distinct orders.

### Burns.

Burns are more frequent in the female wards, but if they occur with men they are usually of a serious nature, caused by boiler explosions, falling into boiling vats, &c. You must have old blankets ready to place them in, and plenty of hot bottles, as the state of collapse is serious. A large surface superficially burnt is more dangerous to life than a more circumscribed area deeply injured. Considerable skill is required in dressing extensive burns. If the case is serious the sister will attend to it herself, and in this, as in all other surgical dressings, you learn by watching skilled hands far better than any amount of study and reading can teach you. It is always important to keep burns from the air, and to accomplish the dressing of a large surface rapidly one nurse should remove the soiled lint and wool whilst another rapidly replaces them with clean. Great care must be taken to avoid all draughts whilst the dressing is going forward. Whilst using the utmost care and gentleness in removing the soiled dressings (a matter causing acute suffering) remember that a hesitating, timid hand only increases the discomfort of the patient; use the utmost gentleness, but be firm and quick in your actions; the quicker the dressing is done the quicker the inevitable suffering is over. Treatment for burns differs greatly; for myself I like them to be very frequently dressed, for though fatigue naturally follows to the patient, I think that evil is counterbalanced by the gain of having the wounds kept in a purer state. Burns at all times smell very much, and you must do your very best to keep them as clean as possible.

### Presentations.

Miss Henrietta Hawkins, who for more than five years was ward sister at the Metropolitan Hospital, Kingsland Road, has upon her recent resignation been the recipient of several testimonials of the esteem and affection felt for her by those who had in any capacity worked with her. Among the most interesting of these were a gold bangle, presented by the nursing staff, and a purse, with an appropriate inscription engraved on the silver mounting, and containing the sum of £28, given by members past and present of the visiting and resident medical staff, the chaplains, and the nurses. The other parting presents to Miss Hawkins took the form of either books or jewellery.

### Post-Graduate Clinics for Nurses.

By a Trained Nurse.

### XXXI.—SUGGESTIONS ON FEEDING.

There is perhaps no one point on which the nurse can more help the patient to enjoy and properly digest his food than by keeping his mouth, teeth, and gums exquisitely and hygienically clean. Nothing destroys appetite more effectually or creates more distaste for food, than a furred and unwholesome mouth. Where this exists constant mopping and rinsing will do much to help a patient's appetite. A pleasant mouth-wash of glycerine, lemon juice, and water, gives a local tonic to the mouth which picks up a patient's keenness for food. It is very refreshing, too, to have the mouth and throat sponged out with an antiseptic mouth-wash; and a few drops of Eau de Cologne added to a little boracic water is delightful, for the cologne covers the somewhat unpleasant taste of the boracic. If applications of a disagreeable or strong nature, such as creosote, have to be applied to a patient's throat, the nurse should try and make the application come midway between meals, and in addition should carefully spray out the mouth with an agreeable mixture some fifteen minutes before she gives food. I have never forgotten the suffering I underwent when ill with diphtheria through the application of iodoform and creosote to the throat. It never occurred to the sister who superintended or to the two trained nurses who took charge of me (I was their only patient, so there was plenty of leisure to figure out the times of treatment) that there was any relation between these nauseating throat applications and my diet. Sometimes ten minutes before a meal my throat would be "insufflated" with iodoform. And it needs very little imagination to conjure up the ghastliness of cocoa and whipped eggs strongly flavoured with creosote or iodoform. This was not only inefficient nursing, but it was bad dieting. The inevitable consequence was a very serious gastric complication, brought about mainly by the nauseating effect of food mixed with two applications whose flavour by no means sets off beef-tea and custard!

It is curious, too, how the use of a feeding-cup seems to take the edge off a patient's desire for food. Personally, I would never, unless under very exceptional circumstances, use anything for a sick patient unable to be raised for feeding but a bent glass tube. It is clean and fresher looking, the patient will take more by this method, and food given thus is infinitely nicer in flavour than when taken from "feeders," as these seem to possess a mysterious tendency to make all food taste alike. Added to which it is practically very difficult to keep the spouts as scrupulously clean as one would wish.

So far as diet is concerned—as in most other branches of her work—the nurse must possess her soul in patience. For there is no denying the fact that sick people are often most unreasonable about food. Not uncommonly they ask for some special dish which needs much complex preparation, and are both surprised and indignant when its evolution is not accomplished in the space of a few minutes. On these occasions a nurse must summon to her aid all the soothing devices of her nature, and it is good exercise for that greatest of all accomplishments in a nurse—the faculty of "resting her patient." This quality is a most valuable adjunct to the sick person's digestion, for a fussy restless person in the sick-room when the patient is taking food is a distinct incentive to dyspepsia. Before and after meals the atmosphere of the sick-room should be calm and restful, and it is a good plan to abstain from giving a patient a really substantial meal when he is labouring under mental or emotional excitement. At such a time it is well to give him a light nourishing "pick-me-up," and to postpone more serious meals until he shall have somewhat quieted down.

At the same time it is important to bear in mind the established fact that irritability and restlessness are very frequently caused by want of food. It is proverbial that "a hungry man is an angry man," and this is far more true when we substitute "exhausted" for "hungry."

Fits of mental excitement are often allayed at once by the simple administration of food. Indeed, we know that the mere mechanical drawing away of blood from the brain to the stomach when food is given, tends to soothe the mind. It is interesting and instructive for the close observer to note how the tired, irritable, and excited lines disappear from a sick person's face as he takes his food and begins to feel the benefit of its digestion. When a patient under my care begins to show signs of excitability and a tendency to talk too much I at once reproach myself with having allowed him to go too long without nourishment. For a starved brain is often most active. On the other hand, the somnolent, half comatose condition of the mind after a thoroughly good meal properly assimilated is an excellent type of a rested condition. And by judicious management we should try to keep the sick under our care in this healthy restful state.

On the same lines of reasoning it always appears to me the greatest possible mistake to argue with a patient about his food. I know many excellent nursing books advise the nurse "to persuade her patient to take that particular food which seems suitable to his condition." But on this point I am unable to agree with some nursing contemporaries. It is a different matter about a patient's medicine—that he must take—or settle the consequences with the doctor. But arguments and persuasions about food are worse than useless. If the patient does not like it—and I would be inclined to use exactly the same argument for a sick child as for a sick adult—that should end the matter. The food must be brought to the needs of the patient—he must not be forced to the food. I could never subscribe to forcing a healthy or a sick child to take food it did not like. To insist on certain food being taken against inclination is bad dietetics for the healthy and bad nursing in the case of the sick person who has food he turns against thrust upon him. Of course, so much latitude is impossible in hospitals, but even there much more range of diet should be provided. But in private practice the patient must be allowed free choice, and a good nurse will rejoice over difficulties to overcome as Alexander rejoiced in the obstacles to his "world conquering."

It is helpful to remember that the palate is the selecting agent for the stomach, and the stomach puts unlimited confidence in the good judgment and capacity of the palate. And what the palate rejects the stomach will digest only in very grudging fashion—if at all. There will be a sulky feeling all the time that something has been forced on it against the better judgment of its constituted gate-keeper. And there is no dietetic foundation in the argument that a distasteful diet is "good for you." It is the department of the nurse to substitute some form of food equally nourishing which the patient will take, if not with pleasure, at least without disgust—and without the tax on the digestion caused by food taken on sufferance. To give food which is not in every way best suited to the patient's needs and tastes is a form of extravagance, for in the attempt to digest unsuitable food the sick person may waste a good deal of valuable strength and power.

### Asylum Attendants: Their Training and Status.

OUR readers will remember that we republished a letter from the *British Medical Journal* of August 21st on the subject of the Medico-Psychological Association and its certificate. A reply at some length appears in the same journal of September 16th, by Mr. Herbert Barraclough, of the Wilts County Asylum, in which he upholds the standard of training required and the value of the examination of the society. If the certificate did not aim at giving asylum attendants the status of mental nurses we might agree with him. His arguments, however, do not appear to us very forcible, because he does not touch the important point, which is this, that it is quite possible that the certificate may be obtained by a course of instruction so theoretical as to render it possible that real dependable practical knowledge of attendance on the sick, which should constitute the right to use the term "nurse," is not necessarily present at all. The certificate does not demand practical experience for a reasonable length of time in a sick ward. We are happy to know that, thanks to the strenuous endeavours of some asylum superintendents, a much more thorough and practical description of training is being introduced into asylums, and that members of superior classes of society are being induced to enter the ranks of asylum attendants. At the Berrywood Asylum, for instance, the female attendants consist principally of the daughters of farmers, medical men, and clergymen, and they have exceptional opportunities for practice in sick nursing. The work at an asylum must of necessity be arduous and especially trying at first, and all the more necessary is it that the moral and intellectual standard should be a high one. The work should be considered as worthy of admiration as is devotion to the bodily sick, if not more so. Much more self-denial, self-control, and singleness of purpose is required of the attendant in an asylum than of a sick nurse. The sick nurse has the interesting and pleasing prospect of seeing the majority of her patients rapidly passing from improvement to cure, and from the majority too she reaps the direct reward of kindly recognition for her labours on their behalf, and opposition is of the rarest occurrence. Then, too, the variety of the work as she passes from ward to ward in the progress of her training relieves the days and weeks of all monotony, and, indeed, to the true nurse time flies by with astonishing rapidity. In a large institution steady promotion follows as a matter of course on successful labours, and when the training school is left behind an immense choice of posts are open to the well-trained competitor, either connected with her own institution or elsewhere, whilst for the private nurse there is still a steady demand, with fair remuneration. With all these inducements it should hardly be looked upon nowadays as an act of heroism for a strong and healthy young woman to take up sick nursing. The halo might more fitly be transferred to the brow of those who with earnest and serious purpose elect to become attendants on the insane. Much trial of the patience, much cheerfulness, tact, singlemindedness, and steadfastness of purpose is required in the really good asylum attendant. And in return, with very limited improvement in the patients, a large amount of ingratitude and even occasional injury has to be faced. She must expect a life of monotony, frequent anxiety, and even disgust, with small remuneration, very limited possibilities of promotion and scant prospect of securing another post of a higher grade in any other institution or private work. The qualities requisite to make a valuable attendant are of a high order. Attendants possessing such qualifications are immensely desired by all enlightened superintendents, and a really useful vocation lies open to members of the better classes. A very short space of time is required to remove the prejudice attached to the position at the present. Pioneers are necessary as they were before the present order of nurses could entirely supplant the original Sairey Gamp class. At Berrywood a good beginning has been made. Not only is the training of a high order, intellectually and practically, but a better tone is observable from year to year, and thus each year the conditions of asylum work are becoming more congenial. The life there is certainly an opening for the good mental nurse, and for the sick nurse who can afford to extend her training, a period passed in the study of mental disease would be a valuable addition to her store of knowledge. When the true status of the asylum attendant is established, we shall lose the confusion of the term "mental nurse" as bearing with it a claim that the mental attendant, with asylum-training only, can also be a sick nurse of the same calibre as the hospital-trained nurse, and we shall on the other hand not accept the sick nurse as a nurse for mental cases unless her training is as thorough as practical experience in an asylum can make it.

### Minor Appointments.

BOROUGH HOSPITAL, WEST BROMWICH. — Two Charge Nurses have been selected to fill these vacant posts at this institution. Their names are Miss Sarah A. Wood and Miss Edith Clarke. The latter was trained at Monsall Hospital, Manchester, and has since temporarily filled the position of sister at the Borough Hospital, Wolverhampton, and has acted as charge nurse at the Ottershaw Hospital, Surrey. Miss Wood was trained at the Isolation Hospital, Watford.

CAMBERWELL WORKHOUSE, DULWICH.—Miss Gertrude Allen has been appointed Night Charge Nurse at the above institution, and Miss Georgina Armitage as Head Day Nurse. Miss Allen received her training at the Leicester Infirmary, where she was afterwards employed as head nurse; and Miss Armitage was trained at the Manchester Royal Infirmary, being subsequently employed there as charge nurse.

MILE END OLD TOWN INFIRMARY.—Miss Jessy S. Parson has been chosen for the post of Assistant Matron at this infirmary. She was trained at King's College Hospital and received maternity training at the Clapham Maternity Hospital, and has held the appointment of staff nurse at King's College Hospital and more recently was head sister of the Piræus Hospital, Athens.

HACKNEY UNION SCHOOLS, BRENTWOOD.—The post of Assistant Matron to this institution has been filled by the appointment of Miss Phœbe Woods. Miss Woods was trained at the Hackney Union Infirmary, and has acted as charge nurse, ward nurse, and assistant nurse at the same institution, having had over nine years' experience.

POPLAR AND STEPNEY SICK ASYLUM, BROMLEY, E.—Miss Mary Franghiadi has been selected to fill the vacancy of Sister at this institution. Miss Franghiadi received her training at St. Bartholomew's Hospital, and at the time of her appointment to the above position was acting as staff nurse at St. Bartholomew's.

METROPOLITAN CONVALESCENT INSTITUTION, WALTON-ON-THAMES.—Miss Beatrice M. Taylor has been appointed Charge Nurse at this institution. She was trained at Ancoats Hospital, Manchester, and has since held an appointment as village nurse, and has also done private nursing.

ROMFORD UNION INFIRMARY.—Miss Jessie McRitchie has been appointed Charge Nurse at this institution. She was trained and employed at the Parochial Hospital, Dundee, and has done private nursing.

DARTMOUTH COTTAGE HOSPITAL.—Miss Elizabeth Jane Clarke has been appointed Charge Nurse at the above institution. Miss Clarke was trained at the Taunton and Somerset Hospital.

### Nursing in Shanghai.

We are sure our readers who remember the departure of the three English nurses last year to start nursing in Shanghai will be interested to hear of the success that has attended their efforts. This is what the sister-in-charge reports to the Shanghai Municipal Council at the beginning of the year:—

"Since the Municipal Nursing Home was started in November, 1896, ten cases have been attended by the nurses and myself, and both the doctors and patients appear to much appreciate this new arrangement made by the Council for their comfort during sickness, by the fact that the demand for nurses has far exceeded the supply even in these early days, when the scheme can hardly be considered in full working order, or to be very generally known.

"Altogether six applications have had to be refused, including one from an outport, and I have heard of several cases of illness requiring nurses, but not actually applying, as it was known that the entire staff was engaged at the time.

"In several of the cases attended one nurse was inadequate for the work and attention demanded by the patient's condition, and a second nurse would have been desirable had there been a sufficiently large staff to admit of this arrangement.

"In consequence also of the pressure of work the nurses have gone from case to case, without any interval between, which would be an unwise proceeding as a general rule.

"A great want would be met if some beds in the home could be added for people who, for some reason, cannot be nursed in their own homes, and the nursing of several patients could be more easily managed by one or two nurses, being under one roof.

"I greatly hope this may shortly be arranged for, and with that, and a larger staff, the new nursing home may be of far greater benefit to the community even than at present, and an undoubted success, while the working expenses of the home would be increased little, if any, and the amount of fees to be collected by the Municipal Council would be much larger."

Since the above report was written the work of the nurses has continued to increase, and the patients they have nursed amounted to 39 early in August. We think that the advantages of trained nursing in Shanghai has been effectually brought home to the residents since the three English nurses went out, for the form chosen for a memorial in that city of the Queen's long reign is to be a nursing institution and training school, to which is to be attached a home for patients. The exact scheme has not yet been entirely agreed upon. There are difficulties in the way. Naturally the general hospital might be looked to as the training ground for the nurses, and indeed from our point of view unless it can be so utilised the training of nurses in Shanghai should not be attempted, seeing that unless the home were to have an equal or nearly equal number of beds, sufficient practical experience could not be gained to turn out an efficiently-trained nurse. But at the present time the General Hospital is in the charge of French nuns, who are not nurses, and unless these excellent women retire in favour of trained nurses no teaching can be available there. No one could deny for the sake of the patients that this would be an advantage. The male patients fare very badly in consequence of their presence, for they may not nurse them, and consequently the male patients must either take in a servant or submit to the tender mercies of coolies. The hospital committee can have but one object, namely, the good of the patients, for whom the hospital exists, and we cannot but believe that they will further a scheme which will place them in the ranks of progress and of public benefactors. An amalgamation of all the forces available to further the cause of the sick is most desirable, and as the Commemoration Committee and the Municipal Council are ready to co-operate, we trust the General Hospital will not hold aloof. The committee have already consented to consider any scheme in detail sent to them, and we look, therefore, to being able to announce a complete scheme of organised nursing at Shanghai before long.

### The Power of Judgment.

By a Matron.

It is an essential point in the career of a successful nurse that she should be capable of judging fairly and rightly. This quality not being one of the strong points of our sex, it is the more necessary that it should be cultivated. Oftentimes a patient's life hangs upon the thread of his nurse's judgment, and an error may mean a fatal delay. A clever doctor is consistent and logical, his professional knowledge is built upon accepted facts and careful deductions, the treatment of his patient is based upon well-conceived theories, the result of wide experience. But with women it is different; their method of calculation is not the same, they fly to results without weighing evidence. To make up for this deficiency in judgment, Nature has endowed her daughters with a strong power of intuition and a swift perception of the drift of vague ideas. These are most valuable attributes in a nurse, but they do not comprise that other quality which makes men more suited for business habits and professional concerns. The power of judgment is especially necessary in those women who take up worldly professions. They will be brought in contact, and must be able to manage conjoint affairs with men whose natural faculties have adapted them to frame and accept certain codes of professional law and etiquette not so easily recognised by the frailer sex.

The acquirement of this faculty should not be forgotten in the training of our nurses and probationers; we should teach them the importance of not being crippled by their prejudices, and help them to cultivate a judicial mind, by which they can look all round a question instead of down the one straight line of their own convictions. Women workers need to be broad minded and large-hearted, they must not allow themselves to be swayed by prejudice or governed by their opinions.

A woman is more likely to become a prey to her prejudices than to her enthusiasms, which are apt to be short-lived, and the old saw still holds good, "a woman convinced against her will is of the same opinion still." But what will be allowed in a woman will not always be tolerated in a nurse, and those nurses who have learnt to grapple with facts rather than with opinions will find that their judgment receives its due share of consideration. Unless that lesson is learnt, they must be content to fall back upon their ready wit and natural intuition, which may possibly both fail them when most needed.

### Verily Mysterious Rays.

It is reported that so wonderful are the properties of some of the more unknown kinds of "X" rays that in the near future if a sick person sends a photograph to the physician he can diagnose the state of the internal organs. Scepticism receives as many hard blows now-a-days on account of its unbelief as used to fall to the lot of religion, but, like the "X" rays themselves, these X Y Z affairs must be demonstrated before they can be accepted. It goes without saying that they, *i.e.*, did they exist, would be most useful. Instead of stethoscope and note-book, the fashionable physician would pay his professional visits armed only with a "kodak," and take a snap-shot of the invalid whilst he exchanged a cheery greeting, and would then depart, promising to send the prescription when he had obtained a "proof." Nurses, too, for the purpose of study, would replace those elegant charts which open in flaps and disclose interior views of the human organism, by an album of cases. We shall doubtless, by-and-bye, hear of an apparatus that photographs the author's book or essay direct from his brain, and, consequently, there will no longer be such a thing as writer's cramp, and the clicking of the typewriter will no longer be heard in the land.

### Male Nurses at the City Hospital Training School, New York.

By A MALE NURSE.

AFTER a Civil Service examination the male nurses become assistants, or probationers, for three months, first in the medical ward of the hospital, then for three months in the surgical ward. After this they work for three months in the throat ward, and last of all spend three months in the venereal ward. Then they pass an examination on what they have learnt. Those that pass then take charge for three months first of the medical ward, then of the surgical ward, then of the throat and the venereal wards. After satisfactorily passing another examination they receive diplomas as graduate male nurses. Each morning they answer to their names in the chapel at six a.m , before they go into the wards; anyone not answering to the roll-call is fined, and he also loses his pass to the city. The work consists of keeping the wards clean and in order, and these are inspected by the steward every morning at nine a.m.

The beds are made by the patients—of course, the convalescent patients—who are taught by the nurse. The nurse has to keep his medicine list on each medical cupboard, and to put a copy on the desk, where is also placed a copy of all stores wanted, and of the special diet ordered by the physician for the special cases. He must enter in a book the physician's directions each morning when the house physician makes his morning rounds, also all changes as to diet and medicine, as directed. He is responsible to the house physician for all he does. He must be kind and patient to all. His salary for the first year is £2 per month, the second £3. After his two years' training he is expected to nurse mental cases for six months. Some of the nurses who were in my class, at least one I know of, after leaving was appointed superintendent of the hospital in Florida, near Jacksonville. After leaving the school and doing about four years' satisfactory private work, a nurse can become a member of the Bureau for Nurses of the New York Academy of Medicine, 17 and 21, West Forty-Three Street, New York, by filling in a printed form of application. One of the questions asked is, Give four names and addresses of patients you have nursed after leaving the hospital, and four physicians' addresses. A special form is sent to the physicians asking them what class the applicant should be in, and what cases he is fitted for. When the superintendent receives all the answers they are put before the committee of the Bureau for Nurses and the Graduates' Club. If the candidate has given satisfaction to physicians and patients a letter is sent to him informing him that he is elected a member of the Bureau for Nurses of the New York Academy of Medicine, and on registration of the fee, which is £1 per year, he is placed on the list. The rule is not to go out for less than 12s. per day, and in some severe cases £1 is charged. If any complaint is made against a nurse he is at once suspended pending an inquiry. The superintendent told me just before I left New York he had over one thousand nurses (male and female) on the list, showing that nurses are not wanting in America.

### Wants and Workers.

[THE attention of correspondents is directed to the fact that "Helps in Sickness and to Health" (Scientific Press, 28 & 29, Southampton Street, Strand, London, W.C.) will enable them promptly to find the most suitable accommodation for difficult or special cases.]

WANTED, a home for a crippled child of seven. The child is delicate. The mother might be able to pay a little. Address Nurse Ryde, Seaside Home for Invalid Children, West Hill, St. Leonards-on-Sea.

NURSE MARY thanks "M.B." very warmly for so kindly sending the bed chair asked for the poor woman suffering from dropsy. It is greatly valued.

### Everybody's Opinion.

[Correspondence on all subjects is invited, but we cannot in any way be responsible for the opinions expressed by our correspondents. No communication can be entertained if the name and address of the correspondent is not given, or unless one side of the paper only is written on.]

### NURSES' HOSTEL, FRANCIS STREET.

"FAIR PLAY" writes: I read with some surprise the report on "The Hostel" in your last week's issue. It struck me that the reporter could not possibly know in what an unfinished state we are still, and what labour, time, and thought it has taken to progress so far with the inmates' wants to be daily attended to whilst the fitting up was going on. I am a private nurse, and have made The Hostel my home between cases for some years. I think I express the general feeling of all in the house when I say that we cannot be too grateful to Miss C. J. Wood. At her own expense and without any outside help, she has carried on for years a home for nurses at Percy Street, giving us full measure both of kindness and comfort in return for a moderate charged sum. And now we greatly rejoice she is still with us, and head of our "new hostel." Our advantages here are great —the table is liberal, the food well cooked, and we have kind friends in managing director, secretary, and housekeeper. We may not be "artistic," but we are business-like and very comfortable, and, despite criticism, very proud of our "new hostel." As to want of ventilation, the idea is simply amusing in regard to a house where Miss C. J. Wood rules.

[In our criticism of the new Nurses' Hostel we distinctly stated that the nurses who used the hostel "expressed themselves thoroughly satisfied with the new arrangements." This is borne out in the above letter. The rest of the facts we mentioned, especially those concerning ventilation, we leave to those competent to judge to form their own conclusion.—ED. *T. H.*]

### THE WORKING OF THE POOR LAW OFFICERS' SUPERANNUATION ACT.

"T. B." writes: Would you kindly give me your advice, by means of THE HOSPITAL "Mirror," as regards the amendment of 1897 to the Poor Law Officers' Superannuation Act of 1896. Having entered on my present engagement on July 3rd, 1897, I naturally thought that, according to the Amendment Act, I was entitled to withdraw from the Fund, and applied for a form the second week in August. No notice being taken I applied again in writing on September 3rd, and received an answer to the effect that as I was an "attendant" at the above home for the "infirm" the Act did not apply to me as it was only for nurses of the sick and insane. I then wrote to the Local Government Board, who informed me that they would not undertake to advise me in the matter. I may be mistaken, but it seems to me that every obstacle is being put in the way of those who wish to withdraw from this unjust Act and every means used to make those who do not understand it join. Most of those to whom I have spoken on the subject appear to have been misled by promises of a rise in salary, &c., and altogether misunderstood the terms on which they agreed to sign for it. As I have not held a poor law appointment before you will see that it was compulsory on my part when I accepted this engagement to submit to the annual deduction. I should be interested to know whether infirm would, or would not, come under the heading of sick. Apologising for troubling you at such length and hoping for a satisfactory answer through the medium of your valuable paper.

[No apology is needed for bringing forward so interesting a point as the working of the Poor Law Superannuation Act. "T. B.'s" letter touches on a real injustice. From it we gather that the Local Government Board have not yet considered whether attendants on the infirm rank as attendants on the sick or not, and as Dr. Downes is away for his holiday some little time must elapse before a definite opinion can be obtained on the subject from the Board. In the meantime information from others in a similar position to "T. B." would be valuable.—ED. *T. H.*]

### For Reading to the Sick.

### HUMILITY.

"Whosoever exalteth himself shall be abased; and he that humbleth himself shall be exalted."

**Verses.**

Take up thy Cross, nor heed the shame,
Nor-let thy foolish pride rebel;
The Lord for thee the Cross endured
To save thy soul from death and hell.

Take up thy Cross, then, in His strength,
And calmly every danger brave;
'Twill guide thee to a better Home,
And lead to victory o'er the grave.
—*C. W. Everett.*

He to the lowly soul
Doth still Himself impart,
And for His dwelling and His throne
Chooseth the poor in heart.—*John Keble.*

I would not have the restless will
That hurries to and fro,
Seeking for some great thing to do
Or secret thing to know;
I would be treated as a child
And guided where I go.

**Reading.**

The way to mount up is to go down. Every step we take downward makes us higher in the Kingdom of Heaven. Do you desire to be great? Make yourself little. There is a mysterious connection between real advancement and self-abasement. God's instruments are poor and despised; they are busied about what the world thinks petty actions, and no one minds them. They are apparently set on no great works, nothing is seen to come of what they do; they seem to fail; they rise by falling. The more they abase themselves the more like they are to one Himself, and the more like to Him the greater must be their power with Him.—*John Henry Newman.*

When thou art bidden of any man to a wedding sit not down in the highest room . . . but go and sit down in the lowest room; that when he that bade thee cometh, he may say unto thee, Go up higher.—*St. Luke* xiv. 8-10.

Here is a rule which extends to whatever we do. It is plain that the spirit of this command leads us, as a condition of being exalted hereafter, to cultivate here all kinds of little humiliations, instead of loving display, putting ourselves forward, seeking to be noticed, being loud or eager in speech, and bent on having our own way; to be content, nay, to rejoice in being made little of, to perform what to the flesh are servile offices, to be patient under calumny; not to argue, not to judge, unless a plain duty comes in, and all this because the Lord has said that such conduct is the very way to be exalted in His presence.—*John Henry Newman.*

### Appointments.

### MATRONS.

St. Albans Hospital and Dispensary.— Miss Fanny Hadfield has been appointed Matron to the above hospital. Miss Hadfield was trained at the Derbyshire Royal Infirmary, and for nine and a half years was matron of the Bridgnorth and South Shropshire Infirmary, and for two years held the appointment of superintendent of the Bolton Private Nurses' Institution.

### Notes and Queries.

**The contents of the Editor's Letter-box have now reached such unwieldy proportions that it has become necessary to establish a hard and fast rule regarding Answers to Correspondents. In future, all questions requiring replies will continue to be answered in this column without any fee. If an answer is required by letter, a fee of half-a-crown must be enclosed with the note containing the enquiry. We are always pleased to help our numerous correspondents to the fullest extent, and we can trust them to sympathise in the overwhelming amount of writing which makes the new rules a necessity. Every communication must be accompanied by the writer's name and address, otherwise it will receive no attention.**

*Society for Training Medical Women for India.*

(391) I believe there is a society or fund which undertakes to fully train medical women in return for service in India. Will you kindly tell me (1) the name of the society or fund; (2) how to get particulars; (3) what the terms are?—*Medical Student.*

The Church of England Zenana Missionary Society, 9, Salisbury Square, Fleet Street, helps women, we believe, to qualify as doctors for work in the mission field. The London School of Medicine for Women, 30, Handel Street, Brunswick Square, W.C., offers a scholarship of £30 a year for four years, subject to an agreement to practise in India, under the Countess of Dufferin's Fund.

*Training in Exchange for Work.*

(392) Could you kindly tell me where I could get training as a nurse in exchange for dispensing service? I have the Pharmaceutical Society's qualification.—*M. W.*

The only way to hear of such a position would be by advertising.

*Borax and Camphor Hair Wash.*

(393) Would you kindly tell me how to make, and the relative quantities of, the old-fashioned borax, camphor, and rosemary hair wash mentioned in answer to question 382 of The Hospital Mirror for September 11th, 1897?—*Night Superintendent.*

Take one quart of soft water (rain, distilled, or artificially softened with anti-calcaire), and, whilst boiling, pour into it an ounce of crushed camphor and the same quantity of borax. Cover the jug until the lotion is cold. Strain, and add half an ounce of spirits of rosemary. Part the hair, rub the roots well with a piece of flannel saturated with the lotion, and dry thoroughly before making the next parting. The drying is important, as the hair is much softer for it. If the hair be dry or brittle, a little fine oil warmed in a saucer and applied to the roots after the wash has been dried off will be found very beneficial.

*District Nursing on a Provident Basis.*

(394) Dr. Jamieson B. Hurry, Abbotsbrook, Reading, writes saying that he "will be glad to hear of any centre where district nursing has been organised on a provident basis, or where a payment is required for the services of a district nurse from all persons, except those actually destitute," and that "a copy of the annual report of any such centre will be welcome."

*General Nursing and Midwifery Lectures.*

(395) Can you tell me where a lady (not a nurse) can attend lectures in London, first on general nursing, second on midwifery? She has been through the St. John Ambulance, and now wants something more advanced.—*Nurse.*

Lectures are given at the Victoria Commemoration Club, 29, Southampton Street, Strand, and at the Trained Nurses' Club, 12, Buckingham Street, Strand. You should write to the secretaries of both clubs at the addresses given, for particulars of the lectures.

*Monthly Nursing.*

(396) I should like to enter a lying-in-hospital for two or three years. Where shall I apply?—*Dorothy.*

Apply to the Matron, City of London Lying-in-Hospital, City Road, E.C. If you are not suited write to us again.

*Crippled Child.*

(397) Can you tell me of a home for a crippled child of seven? The mother might be able to pay a little.—*N. E.*

Write to the Home for Incurable Children, Maida Vale, W., or consult Burdett's "Hospitals and Charities" for a full list of likely institutions for crippled children. We have put your query in "Wants and Workers," as you may get suitable offers.

*Sick Cookery.*

(398) Where can I obtain lessons in sick cookery?—*Brown Bird.*

Write to the Secretary, Victoria Commemoration Club; the Secretary, Trained Nurses' Club, 12, Buckingham Street, Strand; or to Secretary, Royal British Nurses' Association, 17, Old Cavendish Street, W., and enquire as to classes.

*Indian Nursing Service.*

(399) Can you give me particulars of this service? Must I train in a military hospital? I have had one year's training in a general hospital and considerable experience in private nursing.—*P. D.*

You will find all particulars relating to the Indian nursing service in "How to Become a Nurse," by Honnor Morten (Scientific Press, 28, Southampton Street, Strand). Training in a military hospital is unnecessary, but you would stand little chance of selection without a three years' certificate from a hospital.

*Private Ward.*

(400) Can a patient be received into a private ward on payment of a small fee?—*Mikado.*

Several of the London hospitals have wards for private patients. Amongst these are St. Thomas's and the Great Northern Central Hospitals. Write to the hospitals for terms. Burdett's "Hospitals and Charities" gives further particulars.

